# Fætus in Utero Killed by Lightning

**Published:** 1854-01

**Authors:** 


*Fœtus in Utero killed by Lightning.*— Dr. Carithes reports, in a late number of the Southern Medical and Surgical Journal, a case of a woman, 40 years of age, in good health, who was struck with lightning, and on the tenth day after was delivered of a dead child, which, from the appearance, had been so from the time the mother felt the shock.—*From the N. H. Journal of Medicine.*

